# CASE REPORT Successful Breast Reconstruction in a Patient With Epidermolysis Bullosa

**Published:** 2013-01-18

**Authors:** Claudia R. Albornoz, Jane Goldstein, Geoffrey E. Hespe, Virgilio Sacchini, Evan Matros

**Affiliations:** ^a^Plastic and Reconstructive Surgical Service; ^b^Breast Service, Memorial Sloan-Kettering Cancer Center, York Avenue, New York, NY

## Abstract

**Objectives:** Epidermolysis bullosa is a rare skin disorder characterized by blister formation in response to minor trauma as well as extracutaneous manifestations. Details of the surgical history and technical considerations for performing breast reconstruction in a patient with epidermolysis bullosa are discussed. **Method:** The history and details of breast reconstruction in a patient with epidermolysis bullosa are reported. **Result:** A 56-year-old patient with junctional epidermolysis bullosa developed left breast cancer, which was initially treated with lumpectomy. Two years later, a completion total mastectomy was performed for recurrent disease with immediate 2-stage implant-based reconstruction. Nipple reconstruction was completed using a skate flap technique with full-thickness skin graft harvested from the groin region. No blistering, infection, or wound dehiscence was observed. **Conclusions:** Successful immediate implant breast reconstruction is feasible in patients with epidermolysis bullosa. Reconstructive decision-making should be individualized based on the extent and severity of the skin disease.

Epidermolysis bullosa (EB) is a heterogeneous group of hereditary skin diseases characterized by blistering of the skin induced by mild trauma. Blistering results in open wounds that predispose to both scarring and infection.^1^^,^^2^ Some patients have blistering with minimal impairment, whereas others can sustain life-threatening extracutaneous complications such as bowel perforation. Clinical severity of EB is based upon the depth of blistering within the skin. The mildest form of EB, referred to as *EB Simplex*, is due to blisters of the epidermis.^1^^,^^2^

Junctional EB is due to blisters of the lamina lucida, between the epidermis and dermis, whereas in dystrophic EB, blisters occur within dermis.^2^ The most common plastic surgical procedure performed in patients with EB is for release of the mitten hand deformity.^3^ Because of the delicate nature of the skin in patients with EB, surgical procedures can be a challenge requiring special considerations. Herein successful breast reconstruction in a patient with EB is described.

## CASE REPORT

**Figure 1:**
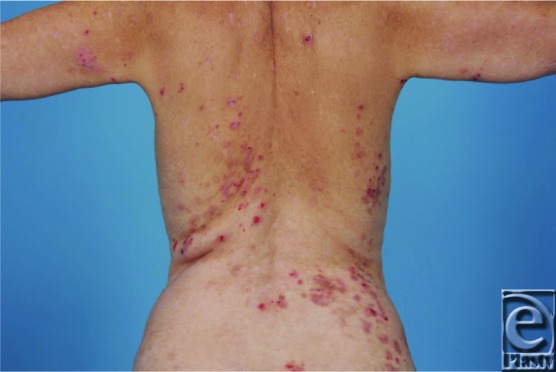
Extent of EB in the current patient.

**Figure 2:**
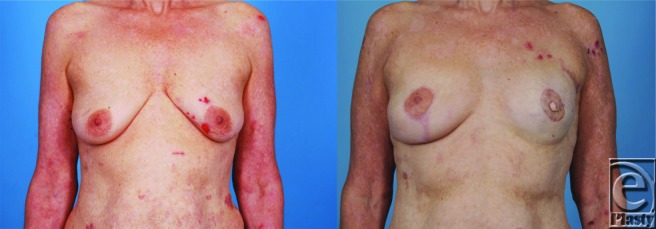
Patient photographs prior to completion mastectomy (*left*) and after staged silicone implant reconstruction (*right*).

A 56-year-old woman with a history of junctional EB with generalized blisters and alopecia presented for evaluation of recurrent left breast cancer (Fig 1). Her initial treatment in 2009 consisted of lumpectomy for ductal carcinoma in situ. She was not offered radiotherapy because of her fragile skin. At the time of her recurrence, in 2010, a total mastectomy was recommended; however, reconstruction was not offered because of EB. The patient came to our institution for a second opinion to assess eligibility for reconstruction. After discussion with EB experts and other clinicians, immediate implant-based breast reconstruction was offered. A tissue expander (TE) was inserted at the time of the total mastectomy with initiation of expansion 1 month thereafter. Exchange of the TE for a silicone implant was performed 3.5 months after the mastectomy. Additional procedures performed to complete reconstruction included the following: autologous fat grafting to improve contour irregularities and rippling, nipple areola reconstruction with a skate flap/full-thickness skin graft, and contralateral mastopexy. The patient had no complications such as blistering, infection, or wound dehiscence. The outcome 24 months following her last procedure is shown in Figure 2.

## DISCUSSION

There is only one report in the literature of breast cancer treatment in a patient with EB. A woman with a 4-cm invasive ductal cancer was successfully treated with breast conservation and adjuvant chemoradiation.^4^ In contrast, the patient reported herein was advised against radiation because of skin fragility, so a completion mastectomy was performed. Breast cancer in patients with EB may be managed with either breast conservation or mastectomy, adjusting treatment on an individual basis. For example, the relative difficulty of surgical manipulation versus the local toxicity of radiotherapy needs to be evaluated in the context of EB severity.

Breast reconstruction in patients with EB involves multiple conceptual considerations. Tissue expansion appears to be safe in all EB variants and has been successfully performed in a patient with dystrophic EB for hand reconstruction.^5^ Patients also need to be informed about the potential risks of bacteremia and implant infection associated with chronic skin breaks. In addition, nipple areola reconstruction requires careful thought. Tattooing is a relative contraindication because of trauma to the fragile skin of patients with EB. Pigmentation can be easily obtained with a full-thickness skin graft.^6^ Feasibility of autologous breast reconstruction is unknown. Technical concerns include the ability of the abdominal wall skin to tolerate closure under tension without blistering or dehiscence; however, the uncomplicated mastopexy performed in the current patient suggests its promise. Microsurgical repair has been successfully performed for hand reconstruction.^5^

Technical barriers when operating on EB patients include anesthesia and difficulty manipulating soft tissues.^7^ Anesthesia is best implemented by use of a lubricated face mask with no tape applied directly to the skin.^4^ Arterial and intravenous catheters should be loosely wrapped with gauze and sutured in the appropriate location. Surgical instruments that could potentially tear the skin, such as forceps, should be substituted for skin hooks or use of hands to perform suturing. The adhesives used for grounding pads of monopolar electrocautery may preclude its utilization. A bipolar or handheld cautery is preferred.

Epidermolysis bullosa has a broad spectrum of cutaneous and extracutaneous manifestations, which need to be factored into reconstructive decision making.^1^^,^^2^^,^^7^ Mild variants such as EB simplex are associated with only occasional blisters with little or no impact on other organ systems.^1^^,^^2^ Junctional EB, the rarest form of the disease, is more severe. Blistering is not only present on the skin but also can involve any mucosal surface. Manifestations include alopecia, pyloric stenosis, and digital flexion deformities.^1^^,^^2^ Dystrophic EB, the most severe form of the disease, is notable for marked skin scarring, which results in the mitten hand deformity.^1^^-^3 Although contracture release can be performed, recurrence is common with significant functional limitation.^7^ Beyond the skin, dystrophic EB has its greatest impact on the gastrointestinal system. Complications include pyloric/esophageal stenosis, constipation, and gastrointestinal blood loss. Corneal ulcerations, joint contractures, muscle atrophy, and laryngeal involvement may also occur.^1^^,^^2^ Some patients survive to adulthood, but most succumb during childhood.^1^ Even within the 3 EB subtypes, there is significant heterogeneity of disease expression. Cases of breast reconstruction are most likely to be expected in patients with milder forms such as simplex and junctional variants.

Implant reconstruction following mastectomy is possible in patients with EB. Special precautions and considerations are required for patients with generalized skin disorder. Knowledge of EB is important, so plastic surgeons can offer reconstruction as well as optimize surgical outcomes.

## References

[1] Lin AN, Carter DM (1989). Epidermolysis bullosa: when the skin falls apart. J Pediatr.

[2] Lin AN, Carter DM (1993). Epidermolysis bullosa. Annu Rev Med.

[3] Mullett F (1998). A review of the management of the hand in dystrophic epidermolysis bullosa. J Hand Ther.

[4] Lyons JA, Schlesinger TE, Smith MD (1999). Successful breast conservation in a patient with epidermolysis bullosa simplex. Breast J.

[5] Whitney TM, Ramasastry S, Futrell JW (1993). Combined tissue expansion and free tissue transfer for reconstruction of the hand in epidermolysis bullosa-associated malignancy. Ann Plast Surg.

[6] Zhong T, Antony A, Cordeiro P (2009). Surgical outcomes and nipple projection using the modified skate flap for nipple-areolar reconstruction in a series of 422 implant reconstructions. Ann Plast Surg.

[7] Ciccarelli AO, Rothaus KO, Carter DM (1995). Plastic and reconstructive surgery in epidermolysis bullosa: clinical experience with 110 procedures in 25 patients. Ann Plast Surg.

